# Detection of *Sarcocystis* spp. and *Toxoplasma gondii* in swine and detection of DNA of these protozoa in tissues and sausages

**DOI:** 10.1590/S1984-29612022049

**Published:** 2022-09-02

**Authors:** Bruna Dias Espindola, Fagner D’ambroso Fernandes, Isac Junior Roman, Gisele Vaz Aguirre Samoel, Roberto Antônio Delgado Barcelos, Alisson Rodrigues Döhler, Sônia de Ávila Botton, Fernanda Silveira Flores Vogel, Luis Antonio Sangioni

**Affiliations:** 1 Laboratório de Doenças Parasitárias – LADOPAR, Departamento de Medicina Veterinária Preventiva – DMVP, Centro de Ciências Rurais – CCR, Universidade Federal de Santa Maria – UFSM, Santa Maria, RS, Brasil

**Keywords:** Swine, *Sarcocystis* spp., Toxoplasma gondii, serology, IFA, PCR, Suínos, *Sarcocystis* spp., Toxoplasma gondii, Sorologia, IFA, PCR

## Abstract

The seroprevalence of *Sarcocystis* spp. and *Toxoplasma gondii* was researched in swine raised in Santa Maria, RS, Brazil. Serum samples from 84 pigs from 31 farms were tested using indirect immunofluorescence assay (IFA) for both agents. Additionally, 53 samples of pork sausages and tissues destined for human consumption, including: salami, sausage, black pudding, heart, tongue, brain, and rib muscle, were submitted to PCR to detect DNA for each agent. The frequency of anti-*Sarcocystis* spp. antibodies was 36.9% (31/84), with titers ranging from 32 to 1024, and 25% (21/84) for anti-*T. gondii* antibodies, with titers ranging from 64 to 2048. *Sarcocystis* spp. and *T. gondii* DNA were detected in 67.9% (36/53) and 13.2% (7/53) of samples, respectively. The presence of antibodies and the detection of DNA from *Sarcocystis* spp., and *T. gondii* suggests that the pigs were infected and may serve as an important reservoir for both parasites. The infection by these protozoa in the swine population is relevant to public health due to their zoonotic potential.

## Resumo

A soroprevalência de *Sarcocystis* spp. e *Toxoplasma gondii* foi pesquisada em suínos criados em Santa Maria, RS, Brasil. Amostras de soro de 84 suínos de 31 fazendas foram testadas pela reação deimunofluorescência indireta (IFA) para ambos os agentes. Adicionalmente, 53 amostras de embutidos suínos e tecidos cárneos destinados ao consumo humano, incluindo: salame, linguiça, morcela, coração, língua, cérebro e músculo da costela foram submetidas à PCR para detecção de DNA para cada agente. A frequência de anticorpos anti-*Sarcocystis* spp. foi de 36,9% (31/84), com títulos variando de 32 a 1.024; e 25% (21/84) para anticorpos anti-*T. gondii*, com títulos variando de 64 a 2048. A presença de DNA de *Sarcocystis* spp. e *T. gondii* foi detectada em 67,9% (36/53) e 13,2% (7/53) das amostras avaliadas, respectivamente. A detecção de anticorpos e DNA de *Sarcocystis* spp. e *T. gondii* sugere que os suínos foram infectados e podem servir como um importante reservatório de ambos os parasitas. A circulação desses agentes na população suína é relevante para a saúde pública devido ao seu potencial zoonótico.

*Sarcocystis* spp. and *Toxoplasma gondii* are obligate intracellular protozoa that have a worldwide distribution and are capable of infecting many animals, including humans, mammals, and birds. These protozoa belong to the phylum Apicomplexa and are heteroxenes; however, *T. gondii* can perform the facultative heteroxenic cycle, when the felid plays the role of intermediate host. The sexual phase occurs in the intestine of definitive hosts, culminating in the production and excretion of oocysts in the feces. In intermediate hosts, tissue cysts are formed during asexual multiplication (Dubey, 2010; Fayer et al., 2015). Transmission of these protozoa to humans can occur through the consumption of raw or undercooked meat or sausages containing tissue cyst with viable bradyzoites, which can cause clinical disease (Cook et al., 2000; Fayer et al., 2015). Additionally, humans can be infected by transplacental transmission, characterizing congenital toxoplasmosis (Dubey, 2010).

A variety of animal species may serve as definitive or intermediate hosts for *Sarcocystis* spp., mainly in farm animals. More than 200 species of *Sarcocystis* were identified; however, *Sarcocystis hominis* and *Sarcocystis suihominis* are zoonotic agents and humans are definitive hosts. Sarcocystosis caused by these agents may present mild clinical signs, however it is usually asymptomatic (Fayer et al., 2015). Swine infection has described in different countries, and three *Sarcocystis* species were identified: *S*. *porcifelis*, *S*. *miescheriana* and *S*. *suihominis* (Kaur et al., 2016; Damriyasa et al., 2004). The infection in pigs may be subclinical, or present mild signs, such as weight loss, anorexia, fever, miscarriage, muscle tremors, and myocarditis (Reiner et al., 2002). Nevertheless, studies involving the detection of this parasite in swine are scarce in Brazil.

Toxoplasmosis caused by *T. gondii* is an important zoonosis. In humans, oral transmission is the most frequent form of infection. Usually, the illness is often asymptomatic; however, in immunocompromised individuals or in cases of transplacental infection, clinical signs may be more severe (Dubey, 2010). In pregnant women, the infection by *T. gondii* may result in vertical transmission leading to miscarriage or fetal death (Cook et al., 2000). In animals, the greatest epidemiological importance of the infection has been verified in swine and small ruminant species. Clinical signs in these species are related to reproductive problems, such as abortion, return to heat, fetal mummification, stillbirths, and increased perinatal mortality. Pigs are more susceptible to *T. gondii* infection due to their omnivorous habits, and tissue cysts have been found in different organs. These animals have been implicated as an important source of *T. gondii* infection in humans (Dubey, 2010).

Considering the importance of both agents, the present study aimed to detect anti-*Sarcocystis* spp. and anti-*T. gondii* antibodies in pigs raised in the municipality of Santa Maria, RS, Brazil. In addition, the presence of DNA from both protozoa in swine tissues and pork derivatives produced in the municipality and intended for human consumption was also investigated.

The municipality of Santa Maria, RS, Brazil has a herd of 3,191 swine, on approximately 689 small properties, intending for family subsistence or local commerce (IBGE, 2017). Population sampling was performed using the non-probabilistic method, for convenience, in the form of a snowball. To carry out this study, 84 pig blood samples were obtained from 31 farms, during May 2020 to March 2021. Conditions of hygienic sanitary management of the properties, presence of other animal species, source of water and food supplies, and sanitation of the facilities were observed in each property. Animals from both sex, different ages and breeds were included in the study. To perform blood collection, animals were manually restrained, and 1 to 5ml of blood was collected from the marginal vein of the ear or jugular, varying according to animal size, and placed in a tube without anticoagulant. Additionally, samples of pork sausages were collected from five open markets and fresh tissue samples from rural producers. A total of 53 samples were obtained, consisting of 32 salamis, nine sausages, one black pudding, six hearts, two brains, two tongues, and one costal muscle. All the samples were obtained from informal production and without veterinary sanitary inspection.

The samples were stored in isothermal boxes and sent to the Laboratório de Doenças Parasitárias (LADOPAR) at Universidade Federal de Santa Maria (UFSM), Santa Maria, Brazil. The blood of each animal was identified and centrifuged to obtain serum. The sausages and tissues were macerated, randomly selected, and divided into two 50 µg aliquots, following the protocol described by Bräunig et al. (2016). Subsequently, the materials were frozen at -20 °C until laboratory analyses. Descriptive statistics were used in this study. All procedures were approved by the Ethics Committee on the Use of Animals at UFSM (3151070220).

Serum samples were analyzed by indirect immunofluorescence assay (IFA) for the detection of anti-*Sarcocystis* spp. (Moon, 1987) and anti-*T. gondii* (Camargo, 1964) antibodies. Briefly, *Sarcocystis* spp. bradyzoites were collected as suggested by Moré et al. (2008) from a naturally infected bovine heart. It was provided by a slaughterhouse inspected by the Municipal Inspection Service. The tachyzoites of *T. gondii* strain RH were obtained from cultivation (Vero cells) (LADOPAR, Santa Maria, Brazil). The antigens, for each agent, were applied in the slides used to perform serological technique. In all reactions, rabbit anti-Pig IgG fluorescein isothiocyanate conjugate (Sigma® Bio Sciences, St Louis, MO, USA) at 1:50 dilution was used as the secondary antibody. Positive and negative controls were used in all reactions. Positive control serum for *Sarcocystis* spp. was obtained by inoculating 1,8 x 10^6^ bradizoites of *Sarcocystis* spp., collected from bovine heart, in a piglet that became seropositive after 21 days. Positive control for *T. gondii* was provided by serum bank stored in LADOPAR. PBS was used as negative controls in IFA assays. The samples were tested in sequential dilutions in base two, from 1:16, until the maximum titer was reached, considering as a positive reaction (cut-off point) animals with anti-*Sarcocystis* spp. and anti-*T. gondii* antibodies equal to or greater than 32 and 64, respectively (Camargo, 1964; Moon, 1987). The reading of the slides was performed under Optiphase INV- 403 epi-fluorescence microscope at a 400X magnification.

To obtain total DNA from all pig samples, the Genomic DNA Purification Kit (Promega®, Madison, USA) was used, following the manufacturer's recommendations, with adaptation in the lysis step, according to Bräunig et al. (2016). The quantity and quality of DNA extraction were evaluated with the aid of a NanoDrop® 1000 spectrophotometer using an absorbance rate of 260/280 nm (Thermo Fisher Scientific). All procedures involving total DNA extraction were carefully performed in order to avoid contamination of the samples. DNA samples were stored at -20 °C until PCR was performed.

To identify the presence of *Sarcocystis* spp. DNA, PCR was performed using the following primers: SARCO F (CGCAAATTACCCAATCCTGA 5'-3 ') and SARCO R (ATTTCTCATAAGGTGCAGGAG 5'-3'), amplifying a fragment of approximately 700bp of the 18S rRNA gene, according to Ferreira et al. (2018). The PCR conditions were as follows: initial heating at 94 °C for 5 min, followed by 35 cycles of denaturation at 94 °C for 45 s, annealing at 60 °C for 30 s, and extension at 72 °C for 50 s, followed by a final extension at 72 °C for 10 min, and 4°C ∝.

To detect *T. gondii* DNA, the primers TOX4 (CGCTGCAGGGAGGAAGACGAAAGTTG 5'-3') and TOX5 (CGCTGCAGACACAGTGCATCTGGATT 5'-3') were used in a PCR reaction, which amplified a repetitive 529 DNA fragment following the methodology described by Homan et al. (2000). In the positive control was used a DNA sample of RH strain tachyzoites from cell culture, provided by the Universidade Estadual de Londrina (UEL). In the negative control ultrapure water was used. The parameters used for DNA amplification were as follows: initial denaturation at 94 °C for 5 min, 35 cycles of denaturation at 94 °C for 30 s, annealing at 63 °C for 45 s, and extension at 72 °C for 45 s, by a final extension at 72 °C for 5 min, and 4°C ∝.

A T100 ™ thermocycler (Bio-Rad^®^, USA) was used to perform all PCR assays. The amplification products were visualized in a UV transilluminator after electrophoresis on a 1.5% agarose gel stained with SYBR® Safe DNA Gel Stain (Invitrogen ™, USA).

The present study was carried out during the COVID-19 pandemic, with limitations in sample collection. Of the 84 samples of sera from pigs tested, 31 (36.9%) and 21 (25%) were positive for anti-*Sarcocystis* spp. and anti-*T. gondii*, antibodies respectively. Serological titers for anti*-Sarcocystis* spp. and anti*-T. gondii* antibodies ranged from 32 to 1024 and from 64 to 2048, respectively (Table 1). Antibodies to the two protozoa were found in 4.7% (4/84) of the animals. Of the 31 farms verified, 67.7% (21/31) and 29% (9/31) had at least one swine positive for *Sarcocystis* spp. and *T. gondii*, respectively, and in 12.9% (4/31) indicating the circulation of both protozoa. Furthermore, among the 53 samples of tissues and pork sausages, 67.9% (36/53) and 13.2% (7/53) were positive for *Sarcocystis* spp. and *T. gondii* DNA, respectively (Table 2), and 11.3% (6/53) were positive for both protozoa.

**Table 1:** Titration of Anti-S*arcocystis* spp. and *Toxoplasma gondii* swine antibodies in the indirect immunofluorescence assay (IFA).

*Sarcocystis* spp.			*Toxoplasma gondii*	
Antibody title	Nº of positive pigs (%)		Antibody title	Nº of positive pigs (%)
32	11 (35,5%)		64	8 (38,1%)
64	7 (22,6%)		128	7 (33,3%)
128	6 (19,4%)		256	1 (4,8%)
256	5 (16,1%)		512	2 (9,5%)
512	1 (3,2%)		1024	2 (9,5%)
1024	1 (3,2%)		2048	1 (4,8%)
Total	31 (100,0%)			21(100,0%)

**Table 2:** Polymerase chain reaction results (PCR) for *Sarcocystis* spp. (18S rRNA) and *Toxoplasma gondii* (529bp) in tissue and sausage samples collected in open markets and direct from producers in the municipality of Santa Maria, Rio Grande do Sul, Brazil.

Samples		*Sarcocystis* spp.		*Toxoplasma gondii*
	n	Nº of positive sample (%)		Nº of positive sample (%)
Salami	32	20 (62.5%)		5 (15.6%)
Sausage	9	5 (55.5%)		1 (16.6%)
Hearts	6	6 (100%)		1 (50%)
Brains	2	1 (50%)		0
Tongues	2	2 (100%)		0
Costal muscle	1	1 (100%)		0
Black pudding	1	1 (100%)		0
Total	53	36 (67.9%)		7 (13.2%)

In relation to the protozoan *Sarcocystis* spp., it is noteworthy that studies evaluating the occurrence of anti-*Sarcocystis* antibodies in swine are limited. In addition, we emphasized that until the end of this study, no data on the prevalence of swine sarcocystosis were found in Brazil.

The lack of standardization of the cutoff point for IFA and the use of different diagnostic techniques in previous studies, difficult comparisons. Using the IFA technique, Moon (1987) detected cross-reactivity between *Sarcocystis* spp. and *T. gondii* until a titer of 16. This result is similar to that reported in other countries using different techniques. In Spain, 43% of the animals analyzed were seropositive using the indirect hemagglutination test (IHA) for detection of anti- *Sarcocystis* spp. antibodies (Pereira & Bermejo, 1988). Damriyasa et al. (2004) used ELISA to evaluate *Sarcocystis* spp. seroprevalence in 2.041 swine females from 230 properties in Germany, and observed that 29% of the animals located in 72% of the properties presented anti-*Sarcocystis* spp. antibodies. In Argentina, 53.33% (IFAT) and 32.08% (ELISA) of the evaluated samples presented seroprevalence, while 81.5% of the properties presented seropositivity for *T. gondii* (Kunic et al., 2022). In the present study, 67.7% of the animals were seropositive for *Sarcocystis* spp.

In all properties evaluated, the pigs were semi-confined and/or free-living, and the sanitary management conditions were precarious. In addition, other animal species, such as dogs, cats, chickens, birds, and cattle were in contact with the swine. Fayer et al. (2015) highlighted the importance of the coexistence of dogs in infection by *S. miescheriana*, as the definitive hosts of this protozoan. The feed of these pigs consisted of horticultural waste and food leftovers from commercial establishments, rations, pasture, and broken grains. The water offered came from sources such as slopes, weir and artesian wells. Studies about the risk factors associated with *Sarcocystis* spp. infection in pigs are scarce in the literature. However, Damriyasa et al. (2004) and Kaur et al. (2016) suggested that management conditions in swine farming and the age of the animals influence the prevalence of infection, due to prolonged exposure to the infective forms of the parasitic agent that are present in the environment and in contaminated feed, especially in animals raised extensively. Furthermore, the severity of the disease is related to the amount of sporocyst ingested by the animal. Although infection in pigs is often subclinical, there is a reduction in weight gain in the affected animals, resulting in economic losses, especially in finishing animals (Reiner et al., 2002).

In the present study, 67.9% of the samples submitted to PCR were positive for *Sarcocystis* spp., demonstrating a high prevalence while compared to previous reports (Pereira & Bermejo, 1988; Damriyasa et al., 2004). In another hand, it is similar to the observed in India, where Kaur et al. (2016) using PCR detected *Sarcocystis* spp. in 72.8% of pig heart destined to human consumption. In China, Huang et al. (2019) using PCR technique, detected *S. miescheriana* and S. *suihominis* in 39.2% and 17.1% swine tissue samples, respectively. The variation in DNA detection in these studies may be related to the extraction of DNA from tissue samples being performed by other protocols, as well as the use of primers with other amplification regions of the genome.

The consumption of raw or undercooked pork meat containing *Sarcocysts* spp. is a risk factor for human infection due to the zoonotic potential. The results of this study did not allow the differentiation of *Sarcocysts* species; however, the presence of anti-*Sarcocystis* spp. antibodies in sera samples, as well as the DNA detection in tissues and sausage shows the participation of these animals in the life cycle of *Sarcocystis* spp.

The detection of *T. gondii* antibodies (26.9%) obtained in this study is in agreement with the reported mean for pigs from subsistence farming in South of Brazil, with 36% (36/100) found in Rio Grande do Sul (Cademartori et al., 2014) and 25.5% (104/408) in Paraná (Millar et al., 2008). The results of the detection of anti-*T. gondii* found in the animals are compatible with the swine rearing system analyzed. Guo et al. (2015) considered that the risk of infection is associated with the extensive swine rearing system, the lack of sanitary management, environmental contamination and the presence of other animal species cohabiting the same environment, including definitive hosts.

In Brazil, prevalence of *T. gondii* infection is lower in industrial farms compared to domestic farms, where animals are raised only for family subsistence (Piassa et al., 2010). A decrease in the infection rate of this protozoan in swine has been observed due to the modernization of production systems and implementation of hygienic sanitary programs in industrial breeding (Guo et al., 2015). The highest titer for anti-*T. gondii* observed in this study was 2048, however 128 and 64 were the most frequent (Table 1). Millar et al. (2008) when testing anti-*T. gondii* antibodies in swine, by IFA, observed a higher frequency of titers of 64, being suggestive of chronic infection, similarly as obtained in the present study.

The presence of protozoa in tissue samples and pork sausages intended for human consumption can have an impact on public health, due to the possibility of human infection (Cook et al. 2000). The detection of *T. gondii* DNA by PCR, found in this study (13.2%), is similar to that reported in Poland by Sroka et al. (2019), obtaining *T. gondii* in 12.2% of pig heart and diaphragm samples. On the other hand, other studies reported higher occurrences of *T. gondii* in swine when compared to our results. Therefore, Aspinall et al. (2002) observed 38% of porcine tissues and sausages in the UK, Bacci et al. (2015) reported 57% of porcine heart samples in Italy and Herrero et al. (2016) demonstrated 73.7% of porcine heart, tongue, and muscle samples in Spain. In Brazil, a prevalence of 27.5% in pork sausages has been reported in São Paulo (Silva et al., 2005) and 39% in Rio Grande do Sul (Costa et al., 2018).

Finally, our results demonstrate the presence of *Sarcocystis* spp. and *T. gondii* in swine and pork tissues and sausages intended for human consumption in the central region of Rio Grande do Sul, Brazil. Therefore, it is emphasized the importance of implementing hygienic and sanitary improvements in non-technified breeding systems in order to minimize the risks of transmission of these agents. Further studies are needed to identify the species of the genus *Sarcocystis* that circulate in swine populations with zoonotic potential.
